# Body habitus in patients with and without bronchiectasis and non-tuberculous mycobacteria

**DOI:** 10.1371/journal.pone.0185095

**Published:** 2017-09-28

**Authors:** Michael D. Schweitzer, Oriana Salamo, Michael Campos, Dean E. Schraufnagel, Ruxana Sadikot, Mehdi Mirsaeidi

**Affiliations:** 1 Section of Pulmonary, Department of Medicine, Miami VA Medical Center, Miami, FL, United States of America; 2 Division of Pulmonary and Critical Care, Department of Medicine, University of Miami, Miami, FL, United States of America; 3 Division of Pulmonary and Critical Care, Department of Medicine, University of Illinois at Chicago, Chicago, IL, United States of America; 4 Division of Pulmonary and Critical Care, Department of Medicine, University of Emory, Atlanta, GA, United States of America; Indian Institute of Technology Delhi, INDIA

## Abstract

**Background:**

Female gender, tall stature, presence of bronchiectasis are associated with pulmonary nontuberculous mycobacterial (NTM) infections. The biologic relationship between the body habitus and NTM infection is not well defined and the body habitus profile of the patients with NTM and concurrent bronchiectasis is completely unknown.

**Methods:**

We conducted a case control study at the Miami VA Healthcare System and the University of Illinois Medical Center on patients with pulmonary NTM infections between 2010 and 2015. We compared pulmonary NTM subjects with and without bronchiectasis. NTM infection was confirmed by using the American Thoracic Society/ Infectious Disease Society of America criteria. Standard radiological criteria were used to define bronchiectasis in chest CT-scan.

**Results:**

Two hundred twenty subjects with pulmonary NTM were enrolled in the study. Sixty six subjects (30%) had bronchiectasis on CT scan of the chest. Subjects in the bronchiectasis group included more women (*p* = 0.002) and were significantly older (*p* = 0.005). Those patients who had bronchiectasis tended to have a significantly lower weight (less than 50kg) and height ≤155 cm (*p <*0.0001 and *p* = 0.018, respectively). Kaplan–Meier analysis confirmed that subjects who had bronchiectasis were shorter and weighed less, after adjusting for gender.

**Conclusions:**

This study defines a new sub-phenotype of NTM subjects with bronchiectasis who tend to be short with lower body weight. Further studies are needed to better understand and define the body habitus profiles of this new sub-phenotype and their clinical implications.

## Introduction

Bronchiectasis is defined as an abnormal dilation of the bronchi, usually irreversible, associated with airway obstruction, chronic productive cough and recurrent infections [1]. It typically begins with airway inflammation almost always after an infectious process [2], linking the release of toxins and immune mediators with the subsequent permanent damage to the respiratory tract anatomy. Moreover, this mechanisms lead to bacterial colonization and recurrent infections, enhancing tissue damage [3]. A broad variety of predisposing factors have been associated with the development of this condition, including infection, congenital defects, genetic predisposition and systemic inflammatory disorders [4]. Bronchiectasis can be further classified into two major groups; cystic fibrosis (CF), that typically occurs in childhood, and non-cystic fibrosis (non-CF) [5]. Previous studies have shown that adult-onset non-CF bronchiectasis usually involved middle aged to elderly women [6].

Non-tuberculous mycobacteria (NTM) are recently recognized as important etiology of pulmonary infections [7]. They are ubiquitous environmental organisms that are frequently isolated from soil and water [8]. Given that NTM infection is not reportable in the United States, it is difficult to determine the accurate prevalence of NTM [9]. It has been reported that the prevalence of NTM has increased in the last decades [10].

Interesting association between NTM and body habitus has been established earlier. In 1992, “Lady Windermere Syndrome” was reported in thin postmenopausal women of European American descent who presented with middle lobe and lingular bronchiectasis and NTM infection, commonly caused by *Mycobacterium avium* complex (MAC) [11].

Later, other studies found similar results and confirmed that tall stature, low body weight, female gender, and elderly were risk factors of NTM infection [12–14]. Among patients with pulmonary NTM, airway diseases including COPD and bronchiectasis are common findings [15].

In our previous work we reported that 37% of patients with NTM had bronchiectasis [15]. Bronchiectasis which is accompanied with small nodular opacities, and a tree-in-bud pattern on CT scan of the chest are associated with a greater probability of isolation of mycobacteria in sputum and bronchial alveolar lavage (BAL) [16]. MAC is the most common isolate in patients with bronchiectasis [15], although other species may be found too [17].

In 2014, our team reported an increased frequency of NTM infections in elderly, thin women [18], but we were unable to show if bronchiectasis was associated with gender, weight, and height in those with pulmonary NTM infections. To our knowledge, the relationship between body habitus and bronchiectasis in patients with NTM infection has not been reported previously.

We hypothesized that patients with bronchiectasis and concurrent NTM infection have different body habitus profile from patients with NTM but without bronchiectasis. We also sought to better understand the association of bronchiectasis with anthropometrics and mycobacterial species.

## Methods

### Study design and data collection

To delineate the body habitus of subjects with NTM and bronchiectasis, we analyzed medical records of patients with confirmed pulmonary NTM from the Miami Veterans Affairs (VA) Healthcare System and University of Illinois at Chicago (UIC), in a retrospective case control study between the years 2010 and 2015. The study was reviewed and accepted by the ethics committee as reflected in the approval numbers 2006–0742 and 574.01. Consent was waived due to the retrospective nature of the study.

Anthropometrics data (age, sex, race, height, weight, and body mass index, BMI), date of onset of symptoms and date of NTM infection diagnosis, clinical symptoms including cough, sputum, hemoptysis, dyspnea, weight loss, smoking habits including current smoking status and number of packs smoked, comorbidities, family history of bronchiectasis, chest imaging findings, sputum microbiology results, history of bronchoscopy and treatment outcomes were collected.

### Criteria and definitions

Subjects 18 years or older were screened if they have International Classification of Diseases ninth revision (ICD-9) code for pulmonary NTM (ICD-9: 031.0). Mycobacterial disease was confirmed if subjects met the clinical, radiologic and microbiologic criteria of American Thoracic Society/Infectious Disease Society of America guidelines for NTM infection [19]. Polymerase chain reaction (PCR) technology was used in both institutes for NTM speciation from respiratory secretion culture, as previously reported [20–22]. Diagnostic criteria for bronchiectasis in chest images included bronchial dilation, bronchial wall thickening, and cylindrical changes [23]. Double reading by radiologist and pulmonologist was used for assurance of accurate diagnosis of bronchiectasis in chest CT.

Outcome was classified as cured, incomplete or no treatment (no treatment offered by physicians). Cured subjects were those who continued antibiotic therapy at least twelve months after sputum conversion to negative. An incomplete outcome was recognized when subjects received at least 6 months treatment but lost to follow up or stopped therapy without physician order. No treatment was defined when physicians decided to not initiate treatment based on individual clinical judgment.

### Statistical analysis

Categorical variables were described as counts and percentages and were tested by the Chi-square test or, if applicable, Fischer’s exact test. Univariate analysis was used to compare differences in demographic and clinical variables between groups with and without bronchiectasis. Normality of distribution of continuous variables was tested by Shapiro-Wilk test. Mann-Whitney U test was used to compare continuous variables because of the majority of variables were not normally distributed.

The Kaplan-Meir estimate curve was used to determine the risk of bronchiectasis for weight and height while adjusting for gender. Curves were compared using the log-rank test. Multivariate logistic regression analysis was performed to identify independent variables that predict the diagnosis of bronchiectasis in subjects with NTM. The model included Anthropometric variables, including age, gender, weight, height and race. The goodness-of-fit of the model was examined by the Hosmer-Lemeshow test. Statistical analysis was performed using IBM SPSS 22.0 statistical software (Armonk, NY).

Two-tailed *p*-values were used and those <0.05 were considered to be statistically significant.

## Results

### Patient characteristics and demographics

Two hundred twenty (62 from Miami VA and 158 from UIC) subjects with pulmonary NTM infection were enrolled in the study. 11 subjects from Miami VA (16.7%) and 51 (33.1%) from UIC had the diagnosis of bronchiectasis. Patients from UIC were older and had more women when compared to patients from Miami VA. Table 1 displays characteristics of enrolled subjects from Miami VA and UIC.

**Table 1:** Comparison of clinical characteristics of subjects with NTM infection at Miami VA and UIC.

Variable	Miami VA	UIC	*p*-value
	**62 (28.1%)**	**158 (71.8%)**	
**Age > 65**	24 (38%)	104 (65%)	<0.0001
**Bronchiectasis**	11 (16.7%)	51 (33.1%)	0.015
**Emphysema**	5 (8.1%)	31 (19.6%)	0.044
**Female**	5 (8.1%)	31 (19.6%)	0.044
**Age Mean (SD) (years)**	61.7 (±13)	67.1 (±14.2)	0.010
**BMI**	23.36 (±5.2)	24 (±7.3)	0.553
**Height Mean (SD) (cm)**	176.5 (±9.1)	164.9 (±8.8)	<0.0001
**Weight Mean (SD) (kg)**	73 (±17)	65.4 (±20.9)	0.015

NTM: non-tuberculous mycobacteria, Miami VA: Miami Veterans Affairs (VA) Healthcare System; UIC: University of Illinois at Chicago; COPD: chronic obstructive pulmonary disease; BMI: body mass index; (SD) standard deviation.

Subjects were grouped as those with bronchiectasis (n = 66, 30%) and without bronchiectasis (n = 154, 70%). The mean (± standard deviation) age of subjects was 69.3 (±13) and 64 (±14.3) years for the bronchiectasis and non-bronchiectasis groups, respectively, with a significant statistical difference between groups (*p* = 0.011).

Majority of subjects with bronchiectasis 66.7% were of European American origin, followed by 7.9% African Americans and 6.3% Asians. In the non-bronchiectasis group, European Americans comprised 60% of the subjects, followed by African Americans (21.2%) and Asians (2.6%). African American race was the only one that showed a significant statistical difference between the bronchiectasis and non-bronchiectasis group (*p* = 0.025).

The bronchiectasis group had more women (47, 71.2%) when compared to the non- bronchiectasis group (74, 48.1%), with a statistical significant difference (*p* = 0.002). 72% of the NTM and bronchiectasis group were 65 years or older when compared to 51.9% in the NTM without bronchiectasis group (*p* = 0.005).

Mean height was 165 cm (±10) and 170 cm (±10) in the bronchiectasis and non-bronchiectasis group, respectively. When subcategorizing subjects according to height less than 155cm, the bronchiectasis group tended to be shorter than those in the non- bronchiectasis group (9, 18% versus 7, 5.8%, respectively) with a significant statistical difference (*p* = 0.018).

The mean weight of subjects with bronchiectasis was 61.9 kg (±18.5) versus 69.9 kg (±20.5) in those without bronchiectasis (*p* = 0.008). Furthermore, weight less than 50 kg, was found in 21 (32.3%) subjects in the bronchiectasis group in contrast to 16 (11%) in the non-bronchiectasis group (*p* <0.0001).

Comparison of characteristics of subjects with NTM and with or without bronchiectasis is listed in Table 2.

**Table 2:** Clinical characteristics of subjects with NTM infection with and without bronchiectasis at Miami VA and UIC.

Variable	NTM with bronchiectasis	NTM without bronchiectasis	*p*-value
**Age Mean (SD) (years)**	69.3 (±13)	64 (±14.3)	0.011
**Age > 65 years**	48 (72%)	80 (51.9%)	0.005
**African American**	5 (7.9%)	32 (21.2%)	0.025^a^
**Asian**	4 (6.3%)	4 (2.6%)	0.207
**Cancer**	11 (16.7%)	28 (18.2%)	0.787
**Cerebrovascular disease**	1 (1.5%)	9 (5.8%)	0.288
**Chronic renal failure**	0 (0%)	5 (3.3%)	0.326
**COPD**	26 (42.6%)	41 (28.9%)	0.56
**Current smoker**	7 (17.5%)	34 (31.5%)	0.091
**Diabetes**	2 (3.1%)	11 (7.6%)	0.21
**Dyspnea**	23 (37.1%)	40 (28.4%)	0.216
**European American**	44 (66.7%)	93 (60%)	0.094
**Ex-smoker**	22 (59.5%)	68 (67.3%)	0.39
**Female gender**	47(71.2%)	74 (48.1%)	0.002
**Fever**	5 (8.1%)	22 (15.7%)	0.141
**Heart failure**	6 (9.2%)	13 (8.9%)	0.939
**Height < 155cm**	9 (18%)	7 (5.8%)	0.018
**Hemoptysis**	20 (32.8%)	33 (23.6%)	0.173
**Hispanic**	1 (1.6%)	13 (8.6%)	0.92
**HIV**	0 (0%)	14 (9.6%)	0.007^a^
**Immunosuppressive therapy (including steroids)**	5 (7.7%)	28 (18.2%)	0.055
**Liver disease**	1 (1.5%)	9 (6.2%)	0.18^a^
**Neurologic disorders**	2 (3%)	15 (9.7%)	0.104
**New onset of cough**	61 (96.8%)	89 (64.5%)	<0.0001
**Night sweats**	13 (21%)	20 (14.4%)	0.245
**Sputum production**	54 (85.7%)	76 (55.9%)	<0.0001
**Weight < 50kg**	21 (32.3%)	16 (11%)	<0.0001
**Weight 10% or below ideal body weight at time of diagnosis**	4 (6.1%)	11 (7.2)	0.077
**Weight loss**	12 (19%)	21 (14%)	0.398
**Weight Mean (SD) (kg)**	61.99 (±18.5)	69.9 (±20.5)	0.008

NTM: non-tuberculous mycobacteria; COPD: chronic obstructive disease; HIV: human immunodeficiency virus.

^a^Fisher’s exact test.

### ICD-9 codes accuracy

A total of 96 subjects were coded for NTM according to ICD-9 codes in the Miami VA cohort. Our review revealed that out of 96 subjects, 17 (22.3%) were wrongly diagnosed or classified, 4 (5.2%) were actually latent tuberculosis patients, and 13 (17.1%) had no microbiological data to support the diagnoses. The accuracy of ICD-9 codes for pulmonary NTM in the Miami VA healthcare system was calculated as low as 65%.

### Microbiological results

Bronchoalveolar lavage (BAL) was performed in 24 (36.4%) and 46 (31.8%) of subjects with and without bronchiectasis, respectively. *Mycobacterium avium* complex was the most commonly isolated species in both groups and significantly higher percentage in the bronchiectasis group (37, 58.7% and 59, 30.9%, *p* = 0.013). *Mycobacterium abscessus* was also isolated in significantly higher number of subjects in bronchiectasis group (17, 27% vs. 19, 12%, *p* = 0.014). *Mycobacterium kansasii* was isolated from 4 (6.3%) and 26 (17.6%) (*p* = 0.03) of NTM subjects with and without bronchiectasis, respectively. *Mycobacterium fortuitum* was isolated from 4 (6.3%) and 25 (16.9%) (*p* = 0.049) of NTM subjects with and without bronchiectasis respectively. Other species, such as *M*. *chelonae*, *M*. *gordonae*, and *M*. *simiae* were isolated without any significant difference between the two groups. More details are shown in Table 3.

**Table 3:** Isolated mycobacteria species in subjects with and without bronchiectasis at Miami VA and UIC.

NTM isolate	NTM with bronchiectasis 66 (30%)	NTM without bronchiectasis 154 (70%)	*p*-value
**MAC**	37 (58.7)	59 (30.9)	**0.013**
***M*. *abscessus***	17 (27)	19 (12)	**0.014**
***M*. *chelonae***	18 (28.6)	27 (18)	0.096
***M*. *fortuitum***	4 (6.3)	25 (16.9)	**0.049**^**a**^
***M*. *gordonae***	6 (9.6)	16 (10.8)	0.780^**a**^
***M*. *kansasii***	4 (6.3)	26 (17.6)	**0.03**^**a**^
***M*. *simiae***	3 (4.8)	4 (2.7)	0.451^**a**^

NTM: non-tuberculous mycobacteria; Miami VA: Miami Veterans Affairs (VA) Healthcare System; UIC: University of Illinois at Chicago; MAC: *Mycobacterium avium* complex.

^a^Fisher’s exact test.

### Comorbidities and clinical characteristics

Cough and sputum production were the most common symptoms in subjects with bronchiectasis (61, 96.8% and 54, 85.7% respectively) compare to those without bronchiectasis (89, 64.5% and 76, 55.9% respectively) (p<0.0001). The presence of comorbidities, including COPD, diabetes mellitus, congestive heart failure, renal disease, chronic liver disease, neurologic disorders and malignancy were not significantly different between the two groups, except for HIV which was significantly higher in those without bronchiectasis (0, 0% and 14, 9.6% *p* = 0.01 in bronchiectasis vs. non-bronchiectasis groups respectively). Table 4 displays chest CT imaging findings of patients with and without bronchiectasis. Of note, all patients had new nodular opacity, but other findings such as emphysema, cavitary lesions, mediastinal lymphadenopathy and pleural effusion were analyzed with no significant differences between bronchiectasis and non-bronchiectasis groups.

**Table 4:** Chest CT imaging findings of patients with and without bronchiectasis at Miami VA and UIC.

NTM isolate	NTM with bronchiectasis 66 (30%)	NTM without bronchiectasis 154 (70%)	*p*-value
**Emphysema**	8 (12)	28 (18)	0.269
**Cavitary lesion**	8 (17.4)	16 (23.9)	0.409
**Lymphadenopathy**	14 (31.8)	16 (18)	0.076
**Pleural effusion**	2 (4.2)	2 (2.8)	0.690

CT: computed tomography; NTM: non-tuberculous mycobacteria, Miami VA: Miami Veterans Affairs (VA) Healthcare System; UIC: University of Illinois at Chicago.

**Fig 1:**
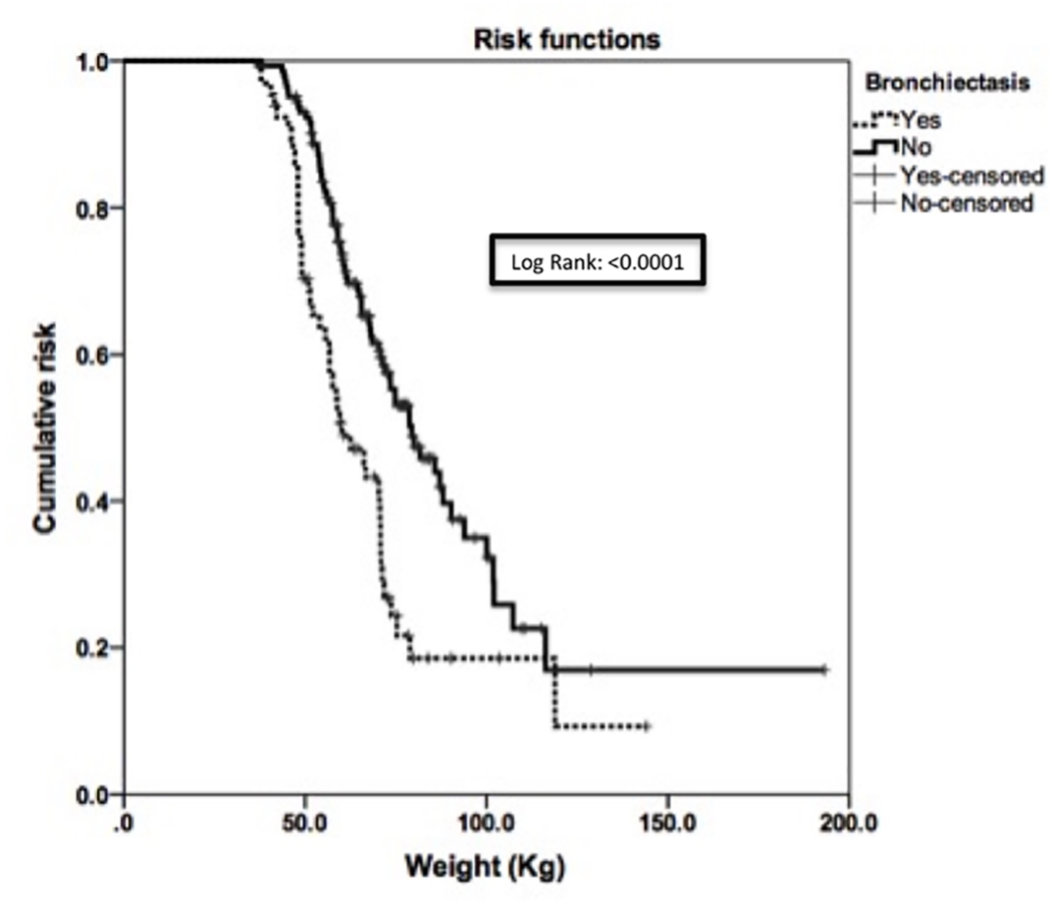
The Kaplan-Meir estimate curve for the cumulative risk according to weight and bronchiectasis, while adjusting for gender.

**Fig 2:**
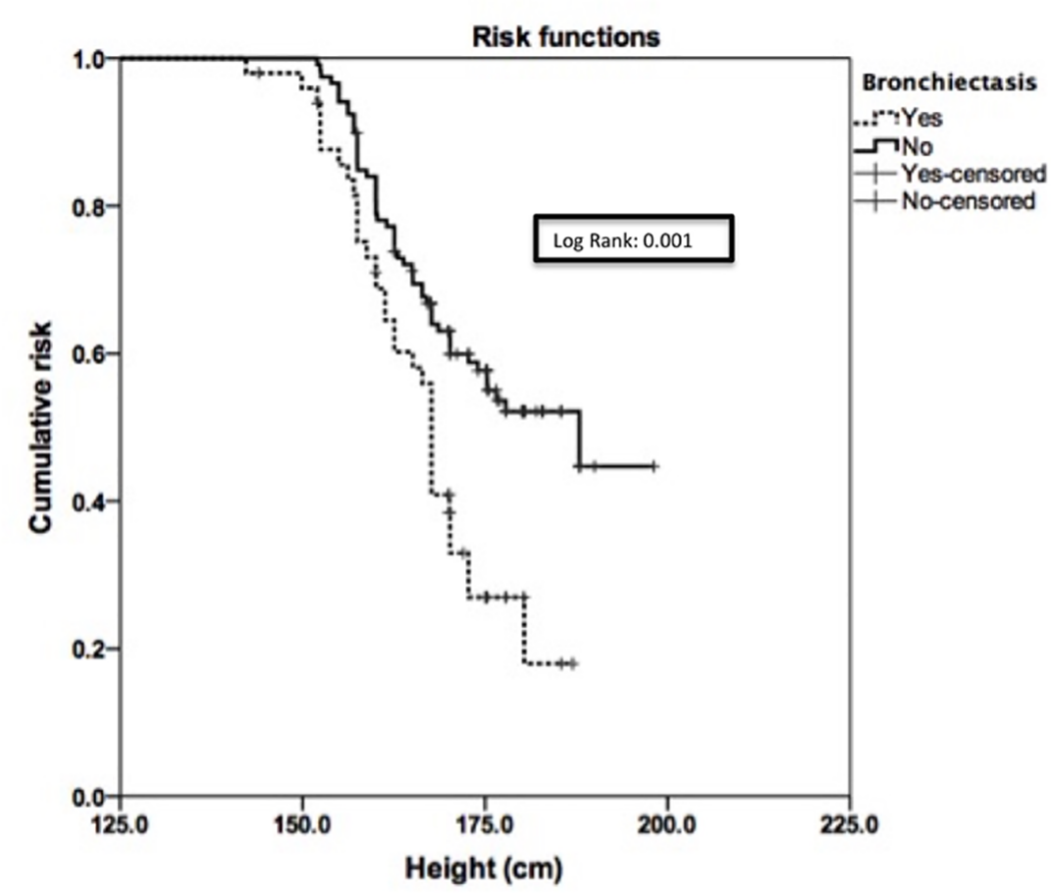
The Kaplan-Meir estimate curve for the cumulative risk according to height and bronchiectasis, while adjusting for gender.

The probability of bronchiectasis in NTM subjects was significantly higher in shorter and lower weighing subjects after adjusting for gender as shown in Figs 1 and 2, respectively.

The multivariable logistic regression model for bronchiectasis in patients with NTM is depicted in Table 5. Among anthropometric variables, age greater than 65 years, weight and African American race were significantly associated with finding bronchiectasis in the chest CT of subjects with confirmed pulmonary NTM.

**Table 5:** Clinical and bacteriological features of subjects with dual NTM infection and bronchiectasis at Miami VA and UIC on multivariate analysis.

Variable	*p*-value, OR (95% CI)
**Age >65 years**	0.006, 3.1 (1.37–6.90)
**African American race**	0.076, 0.335 (0.10–1.12)
**Female**	0.170, 1.8 (0.77–4.12)
**Height**	0.57, 1.02 (0.97–1.07)
**Weight**	0.005, 1.04 (1.01–1.07)

NTM: non-tuberculous mycobacteria; Miami VA: Miami Veterans Affairs (VA) Healthcare System; UIC: University of Illinois at Chicago; OR: odds ratio; CI: confidence interval.

### Clinical outcome

Clinical outcome was found for 12 out of 66 subjects with bronchiectasis. Out of 12, 3 (25%) were cured, 4 (33.33%) had an incomplete outcome, and 5 (41.66%) had no treatment offered by physicians. Clinical outcome was found in 45 out of 154 subjects in non-bronchiectasis group. 14 (31.11%) had a satisfactory outcome, considered them as cured, 25 (55.55%) defined as incomplete outcome, and 6 (13.33%) had no treatment offered by physicians.

## Discussion

Our study reaffirms the close relationship between NTM and bronchiectasis. Bronchiectasis was more frequent in elderly women (age >65 years) with pulmonary NTM as previously reported [24]. *Mycobacterium avium* complex was the most common species isolated from bronchiectasis group. In the US, MAC has been repeatedly reported as the most common bacteria associated with pulmonary NTM [25, 26]. We found significantly higher frequency of MAC in subjects with bronchiectasis in the univariate analysis.

Although the relationship between NTM and bronchiectasis is well established, many questions on this association remained unanswered. Does bronchiectasis increase susceptibility to NTM? Does NTM contribute to the pathogenesis of bronchiectasis? Our study suggests that both scenarios could be true. We, and others, have previously shown that NTM susceptibility increases in elderly women with bronchiectasis [24]. The reasons for the association are not clearly defined but have been speculated. It has been postulated that it might be due to breakdown of the bronchial epithelium in bronchiectasis that subsequently allows NTM to take up residence, grow, and enhance the structural deformities of bronchiectasis. The molecular mechanisms and the sequence remain to be discovered.

Another unsolved puzzle is the reason for high prevalence of NTM in elderly females. It has been suggested that estrogen plays a protective role in NTM infection [27]. It is possible that in menopause women, estrogen protection effect disappears and consequently the risk for NTM increases [27]. Another plausible explanation for higher susceptibility to NTM in aged women might be because of decreased serum concentration of macrophage colony-stimulating factor and consequently the low macrophage activity [28]. Probably there are several other factors may play a role in gender susceptibility to NTM that will be uncovered with further investigations [24].

COPD was the most common comorbidity in bronchiectasis and non-bronchiectasis groups. No other comorbidity, except for HIV, demonstrated any association between pulmonary NTM and bronchiectasis. Interestingly, none of the HIV subjects had concurrent bronchiectasis in our study.

The presence of bronchiectasis was higher in shorter and thinner individuals. To our best knowledge, this finding has not been reported previously. Chen and Iseman reported that slender, older women are more susceptible to NTM, however, they did not separate out the presence of bronchiectasis in their review [13].

A common feature of bronchiectasis is malnutrition [29]. Malnutrition causes short height for the age, secondary to slowing skeletal growth, as well as low weight in children [30]. In adults, malnutrition is often related to low weight, sparing any effect in the height. In our study, the multivariate logistic regression analysis identified the weight (not height) as an independent risk factor for finding bronchiectasis in chest image, suggesting a relationship between adulthood malnutrition with bronchiectasis in subjects with NTM infection. In particular, it would be interesting to determine if this is related to Vitamin D deficiency. We would suggest further investigation on association of malnutrition and bronchiectasis in adults.

In our study, the proportion of NTM subjects without bronchiectasis was greater than reported in other studies. Kim *et al* [12] found that 95% of subjected referred to their clinic were females and > 90% had concurrent bronchiectasis. Our data might be skewed due to reviewing Veterans population, which has dominantly men and high incidence of COPD [31, 32]. Other limitation of our analysis includes its retrospective nature and ICD-9 accuracy, as discussed earlier.

In conclusion, this study demonstrates that subjects with bronchiectasis and pulmonary NTM infections have a specific body habitus. Whether these associations are causal or result of the disease will need further investigation. Future studies are also needed to determine if there are association between some genetic polymorphisms in immune genes and increased susceptibility to NTM in this body habitus, or environmental factors such as malnutrition and vitamin deficiency paly a role in this susceptibility.

## References

[1] Pappalettera M, Aliberti S, Castellotti P, Ruvolo L, Giunta V, Blasi F. Bronchiectasis: an update. Clin Respir J. 2009;3(3):126–34. doi: 10.1111/j.1752-699X.2009.00131.x . 20298395 10.1111/j.1752-699X.2009.00131.x

[2] Morrissey BM. Pathogenesis of bronchiectasis. Clin Chest Med. 2007;28(2):289–96. doi: 10.1016/j.ccm.2007.02.014 . 17467548 10.1016/j.ccm.2007.02.014

[3] Cole PJ. Inflammation: a two-edged sword—the model of bronchiectasis. Eur J Respir Dis Suppl. 1986;147:6–15. . 3533593

[4] Shoemark A, Ozerovitch L, Wilson R. Aetiology in adult patients with bronchiectasis. Respir Med. 2007;101(6):1163–70. doi: 10.1016/j.rmed.2006.11.008 . 17223027 10.1016/j.rmed.2006.11.008

[5] Strausbaugh SD, Davis PB. Cystic fibrosis: a review of epidemiology and pathobiology. Clin Chest Med. 2007;28(2):279–88. doi: 10.1016/j.ccm.2007.02.011 . 17467547 10.1016/j.ccm.2007.02.011

[6] Pasteur MC, Helliwell SM, Houghton SJ, Webb SC, Foweraker JE, Coulden RA, et al An investigation into causative factors in patients with bronchiectasis. Am J Respir Crit Care Med. 2000;162(4 Pt 1):1277–84. doi: 10.1164/ajrccm.162.4.9906120 . 11029331 10.1164/ajrccm.162.4.9906120

[7] Mirsaeidi M, Farshidpour M, Ebrahimi G, Aliberti S, Falkinham JO, 3rd. Management of nontuberculous mycobacterial infection in the elderly. Eur J Intern Med. 2014;25(4):356–63. doi: 10.1016/j.ejim.2014.03.008 ; PubMed Central PMCID: PMCPMC4067452. 24685313 10.1016/j.ejim.2014.03.008 PMC4067452

[8] Velayati AA, Farnia P, Mozafari M, Mirsaeidi M. Nontuberculous Mycobacteria Isolation from Clinical and Environmental Samples in Iran: Twenty Years of Surveillance. Biomed Res Int. 2015;2015:254285 doi: 10.1155/2015/254285 ; PubMed Central PMCID: PMCPMC4477424. 26180788 10.1155/2015/254285 PMC4477424

[9] Winthrop KL, Baxter R, Liu L, McFarland B, Austin D, Varley C, et al The reliability of diagnostic coding and laboratory data to identify tuberculosis and nontuberculous mycobacterial disease among rheumatoid arthritis patients using anti-tumor necrosis factor therapy. Pharmacoepidemiol Drug Saf. 2011;20(3):229–35. doi: 10.1002/pds.2049 ; PubMed Central PMCID: PMCPMC4094092. 21351303 10.1002/pds.2049 PMC4094092

[10] Adjemian J, Olivier KN, Seitz AE, Holland SM, Prevots DR. Prevalence of nontuberculous mycobacterial lung disease in U.S. Medicare beneficiaries. Am J Respir Crit Care Med. 2012;185(8):881–6. doi: 10.1164/rccm.201111-2016OC ; PubMed Central PMCID: PMCPMC3360574. 22312016 10.1164/rccm.201111-2016OC PMC3360574

[11] Reich JM, Johnson RE. Mycobacterium avium complex pulmonary disease presenting as an isolated lingular or middle lobe pattern. The Lady Windermere syndrome. Chest. 1992;101(6):1605–9. . 1600780 10.1378/chest.101.6.1605

[12] Kim RD, Greenberg DE, Ehrmantraut ME, Guide SV, Ding L, Shea Y, et al Pulmonary nontuberculous mycobacterial disease: prospective study of a distinct preexisting syndrome. Am J Respir Crit Care Med. 2008;178(10):1066–74. doi: 10.1164/rccm.200805-686OC ; PubMed Central PMCID: PMCPMC2720143. 18703788 10.1164/rccm.200805-686OC PMC2720143

[13] Chan ED, Iseman MD. Slender, older women appear to be more susceptible to nontuberculous mycobacterial lung disease. Gend Med. 2010;7(1):5–18. doi: 10.1016/j.genm.2010.01.005 . 20189150 10.1016/j.genm.2010.01.005

[14] Okumura M, Iwai K, Ogata H, Ueyama M, Kubota M, Aoki M, et al Clinical factors on cavitary and nodular bronchiectatic types in pulmonary Mycobacterium avium complex disease. Intern Med. 2008;47(16):1465–72. . 18703856 10.2169/internalmedicine.47.1114

[15] Mirsaeidi M, Hadid W, Ericsoussi B, Rodgers D, Sadikot RT. Non-tuberculous mycobacterial disease is common in patients with non-cystic fibrosis bronchiectasis. Int J Infect Dis. 2013;17(11):e1000–4. Epub 2013/05/21. doi: 10.1016/j.ijid.2013.03.018 . 23683809 10.1016/j.ijid.2013.03.018 PMC4472485

[16] Koh WJ, Lee KS, Kwon OJ, Jeong YJ, Kwak SH, Kim TS. Bilateral bronchiectasis and bronchiolitis at thin-section CT: diagnostic implications in nontuberculous mycobacterial pulmonary infection. Radiology. 2005;235(1):282–8. doi: 10.1148/radiol.2351040371 . 15703315 10.1148/radiol.2351040371

[17] Bonaiti G, Pesci A, Marruchella A, Lapadula G, Gori A, Aliberti S. Nontuberculous Mycobacteria in Noncystic Fibrosis Bronchiectasis. Biomed Res Int. 2015;2015:197950 doi: 10.1155/2015/197950 ; PubMed Central PMCID: PMCPMC4461751. 26106603 10.1155/2015/197950 PMC4461751

[18] Mirsaeidi M, Machado RF, Garcia JG, Schraufnagel DE. Nontuberculous mycobacterial disease mortality in the United States, 1999–2010: a population-based comparative study. PLoS One. 2014;9(3):e91879 doi: 10.1371/journal.pone.0091879 ; PubMed Central PMCID: PMCPMC3954860. 24632814 10.1371/journal.pone.0091879 PMC3954860

[19] Griffith DE, Aksamit T, Brown-Elliott BA, Catanzaro A, Daley C, Gordin F, et al An official ATS/IDSA statement: diagnosis, treatment, and prevention of nontuberculous mycobacterial diseases. Am J Respir Crit Care Med. 2007;175(4):367–416. doi: 10.1164/rccm.200604-571ST . 17277290 10.1164/rccm.200604-571ST

[20] Saini V, Raghuvanshi S, Khurana JP, Ahmed N, Hasnain SE, Tyagi AK, et al Massive gene acquisitions in Mycobacterium indicus pranii provide a perspective on mycobacterial evolution. Nucleic Acids Res. 2012;40(21):10832–50. doi: 10.1093/nar/gks793 ; PubMed Central PMCID: PMCPMC3505973. 22965120 10.1093/nar/gks793 PMC3505973

[21] Rahman SA, Singh Y, Kohli S, Ahmad J, Ehtesham NZ, Tyagi AK, et al Comparative analyses of nonpathogenic, opportunistic, and totally pathogenic mycobacteria reveal genomic and biochemical variabilities and highlight the survival attributes of Mycobacterium tuberculosis. MBio. 2014;5(6):e02020 doi: 10.1128/mBio.02020-14 ; PubMed Central PMCID: PMCPMC4222108. 25370496 10.1128/mBio.02020-14 PMC4222108

[22] Singh Y, Kohli S, Sowpati DT, Rahman SA, Tyagi AK, Hasnain SE. Gene cooption in mycobacteria and search for virulence attributes: comparative proteomic analyses of Mycobacterium tuberculosis, Mycobacterium indicus pranii and other mycobacteria. Int J Med Microbiol. 2014;304(5–6):742–8. doi: 10.1016/j.ijmm.2014.05.006 . 24951307 10.1016/j.ijmm.2014.05.006

[23] Hartman TE, Primack SL, Lee KS, Swensen SJ, Muller NL. CT of bronchial and bronchiolar diseases. Radiographics. 1994;14(5):991–1003. doi: 10.1148/radiographics.14.5.7991828 . 7991828 10.1148/radiographics.14.5.7991828

[24] Mirsaeidi M, Sadikot RT. Gender susceptibility to mycobacterial infections in patients with non-CF bronchiectasis. Int J Mycobacteriol. 2015;4(2):92–6. doi: 10.1016/j.ijmyco.2015.05.002 ; PubMed Central PMCID: PMCPMC4470303. 26097805 10.1016/j.ijmyco.2015.05.002 PMC4470303

[25] Mirsaeidi M, Vu A, Leitman P, Sharifi A, Wisliceny S, Leitman A, et al A Patient-Based Analysis of the Geographic Distribution of Mycobacterium avium complex, Mycobacterium abscessus, and Mycobacterium kansasii Infections in the United States. Chest. 2017;151(4):947–50. doi: 10.1016/j.chest.2017.02.013 . 28390637 10.1016/j.chest.2017.02.013

[26] Johnson MM, Odell JA. Nontuberculous mycobacterial pulmonary infections. J Thorac Dis. 2014;6(3):210–20. doi: 10.3978/j.issn.2072-1439.2013.12.24 ; PubMed Central PMCID: PMCPMC3949190. 24624285 10.3978/j.issn.2072-1439.2013.12.24 PMC3949190

[27] Han XY, Tarrand JJ, Infante R, Jacobson KL, Truong M. Clinical significance and epidemiologic analyses of Mycobacterium avium and Mycobacterium intracellulare among patients without AIDS. J Clin Microbiol. 2005;43(9):4407–12. doi: 10.1128/JCM.43.9.4407-4412.2005 ; PubMed Central PMCID: PMCPMC1234053. 16145084 10.1128/JCM.43.9.4407-4412.2005 PMC1234053

[28] Kamada M, Irahara M, Maegawa M, Ohmoto Y, Takeji T, Yasui T, et al Postmenopausal changes in serum cytokine levels and hormone replacement therapy. Am J Obstet Gynecol. 2001;184(3):309–14. doi: 10.1067/mob.2001.109940 . 11228479 10.1067/mob.2001.109940

[29] King PT. The pathophysiology of bronchiectasis. Int J Chron Obstruct Pulmon Dis. 2009;4:411–9. ; PubMed Central PMCID: PMCPMC2793069. 20037680 10.2147/copd.s6133 PMC2793069

[30] Van de Poel E, Hosseinpoor AR, Speybroeck N, Van Ourti T, Vega J. Socioeconomic inequality in malnutrition in developing countries. Bull World Health Organ. 2008;86(4):282–91. doi: 10.2471/BLT.07.044800 ; PubMed Central PMCID: PMCPMC2647414. 18438517 10.2471/BLT.07.044800 PMC2647414

[31] Luna Diaz LV, Iupe I, Zavala B, Balestrini KC, Guerrero A, Holt G, et al Improving adherence to alpha-1 antitrypsin deficiency screening guidelines using the pulmonary function laboratory. Int J Chron Obstruct Pulmon Dis. 2017;12:2257–9. doi: 10.2147/COPD.S143424 ; PubMed Central PMCID: PMCPMC5546190. 28814853 10.2147/COPD.S143424 PMC5546190

[32] Medrek SK, Sharafkhaneh A, Spiegelman AM, Kak A, Pandit LM. Admission for COPD Exacerbation Is Associated with the Clinical Diagnosis of Pulmonary Hypertension: Results from a Retrospective Longitudinal Study of a Veteran Population. COPD. 2017:1–6. doi: 10.1080/15412555.2017.1336209 . 28715281 10.1080/15412555.2017.1336209

